# Exploring Reusability of Disposable Face Masks: Effects of Disinfection Methods on Filtration Efficiency, Breathability, and Fluid Resistance

**DOI:** 10.1002/gch2.202100030

**Published:** 2021-07-28

**Authors:** Jye Yng Teo, Jessica Kng, Balamurugan Periaswamy, Songlin Liu, Poh‐Chong Lim, Chen Ee Lee, Ban Hock Tan, Xian Jun Loh, Xiping Ni, Daniel Tiang, Guangshun Yi, Yee Yian Ong, Moi Lin Ling, Wei Yee Wan, Hei Man Wong, Molly How, Xiaohui Xin, Yugen Zhang, Yi Yan Yang

**Affiliations:** ^1^ Institute of Bioengineering and Bioimaging 31 Biopolis Way, The Nanos #07‐01 Singapore 138669 Singapore; ^2^ Institute of Materials Research and Engineering 2 Fusionopolis Way, Innovis, #08‐03 Singapore 138634 Singapore; ^3^ Singapore Health Services Pte Ltd 10 Hospital Boulevard, Level 19 SingHealth Tower Singapore 168582 Singapore; ^4^ Infectious Diseases Singapore General Hospital Outram Road Singapore 169608 Singapore; ^5^ Infection Prevention & Epidemiology Singapore General Hospital Outram Road Singapore 169608 Singapore

**Keywords:** breathability, disinfection, disposable face masks, filtration efficiency, fluid resistance

## Abstract

To curb the spread of the COVID‐19 virus, the use of face masks such as disposable surgical masks and N95 respirators is being encouraged and even enforced in some countries. The widespread use of masks has resulted in global shortages and individuals are reusing them. This calls for proper disinfection of the masks while retaining their protective capability. In this study, the killing efficiency of ultraviolet‐C (UV‐C) irradiation, dry heat, and steam sterilization against bacteria (*Staphylococcus aureus*), fungi (*Candida albicans*), and nonpathogenic virus (*Salmonella virus P22*) is investigated. UV‐C irradiation for 10 min in a commercial UV sterilizer effectively disinfects surgical masks. N95 respirators require dry heat at 100 °C for hours while steam treatment works within 5 min. To address the question on safe reuse of the disinfected masks, their bacteria filtration efficiency, particle filtration efficiency, breathability, and fluid resistance are assessed. These performance factors are unaffected after 5 cycles of steam (10 min per cycle) and 10 cycles of dry heat at 100 °C (40 min per cycle) for N95 respirators, and 10 cycles of UV‐C irradiation for surgical masks (10 min per side per cycle). These findings provide insights into formulating the standard procedures for reusing masks without compromising their protective ability.

## Graphical abstract

The ongoing coronavirus pandemic has resulted in global shortages of disposable face masks, prompting them to be reused. To ensure safe reuse, masks undergo various disinfection methods including ultraviolet‐C irradiation, dry heat, and steam sterilization. Particularly, five cycles of steam treatment (10 min per cycle) do not impair the filtration capabilities, breathability, and fluid resistance property of N95 respirators.

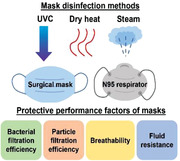

## 1 Introduction

The COVID‐19 is a highly infectious disease caused by the severe acute respiratory syndrome coronavirus 2 (SARS‐CoV‐2).^[^ ^1^ ^]^ Since its first emergence in Wuhan, China, in December 2019, the virus has been detected in nearly every country and a pandemic was declared by the World Health Organization (WHO) in March 2020. As of 3 May 2021, the WHO reported that at least 146 million people worldwide have contracted COVID‐19 with more than 3.1 million confirmed deaths.^[^ ^2^ ^]^ Through genomic sequencing and phylogenetic analysis, it was discovered that COVID‐19 has a large resemblance to two bat‐derived SARS‐like coronaviruses with 88% similarity.^[^ ^3^, ^4^ ^]^ While COVID‐19 has a low mortality rate, it is highly transmissible and infectious.^[^ ^4^ ^]^ In human‐to‐human transmission, the virus is most commonly disseminated by respiratory droplets released through coughing, sneezing and talking.^[^ ^5^ ^]^ The widespread global infection rate of the virus can be attributed to its long incubation period (ranging 2–14 days) and transmission from asymptomatic hosts.^[^ ^4^, ^5^, ^6^ ^]^

To curb further spread of the virus, the use of protective face masks such as surgical masks and N95 respirators was recommended as a preventive measure.^[^ ^6^, ^7^ ^]^ The U.S. Food and Drugs Administration (FDA) defines a surgical mask as a loose‐fitting, single‐use disposable device that covers the nose and mouth of the wearer and serves as a physical barrier to both fluid and large particulate contaminants.^[^ ^8^ ^]^ These may be used by the public as well as healthcare personnel during surgical procedures. However, they do not provide a complete protection due to the loose fit between the face and mask. On the other hand, N95 respirators are designed to seal around the nose and mouth of the wearer and filter sub‐micron sized airborne particles efficiently.^[^ ^8^ ^]^ They are intended for one‐time use by healthcare personnel and must be fit tested.^[^ ^9^ ^]^ NIOSH‐approved N95 respirators are evaluated and certified by the U.S. National Institute of Occupational Safety and Health (NISOH) to have a particulate filtration efficiency of at least 95%. Surgical N95 respirators are both approved by NIOSH as an N95 respirator and cleared by FDA as a surgical mask.^[^ ^8^, ^9^ ^]^ Surgical masks and N95 respirators are typically made of multiple nonwoven fabric layers.^[^ ^10^ ^]^ The inner layer (wearer side) is designed to contain bodily fluids such as sweat of the wearer; the middle filter layer is to prevent particles and pathogens of certain cut‐off size from entering and exiting the mask; the outer layer is fluid resistant to limit the penetration of fluids from the immediate environment.^[^ ^11^ ^]^

While there is limited scientific evidence that wearing masks by healthy individuals can prevent them from getting infected with COVID‐19 in the wider community,^[^ ^7^, ^12^ ^]^ the use of masks has shown to reduce the spread of respiratory droplets released in the air during a cough or a sneeze.^[^ ^13^ ^]^ This may in turn greatly reduce the emission of viral particles into the environment and mitigate the transmission of COVID‐19. Following the successful demonstration by China in limiting the spread of the virus with the use of face masks, many countries across Asia and Europe have mandated wearing masks in public places.^[^ ^14^, ^15^ ^]^ While recommendations on mask wearing vary across countries, the massive surge in the demand for masks has resulted in global shortages. Health experts warned the possibility of supply shortages persisting into year 2021 and longer,^[^ ^16^ ^]^ triggering researchers to look into sustainable materials such as cellulose for mask manufacturing.^[^ ^17^, ^18^, ^19^, ^20^ ^]^

To mitigate the current shortages of masks, the FDA has allowed manufacturers to market face masks without having to submit a 510(K) premarket notification under specific requirements of the Enforcement Policy.^[^ ^9^ ^]^ The issuance of Emergency Use Authorization (EUA) for certain face masks by FDA has also increased the availability of masks to healthcare personnel and the public.^[^ ^9^ ^]^ Additionally, public health organizations such as the U.S. Centers for Disease Control and Prevention (CDC) and European Centre for Disease Prevention and Control (ECDC) have recommended conservation strategies including the possibility of reusing disposable masks after decontamination.^[^ ^21^, ^22^ ^]^

With the support of governmental agencies, research labs and healthcare organizations across the globe have dedicated efforts to investigate the effectiveness of common disinfection methods on disposable face masks including ultraviolet‐C (UV‐C) irradiation, dry heating and moist heating. These research papers typically focused on one disinfection method testing a spectrum of microbes with only some evaluating the performance of the disinfected masks over number of cycles.^[^ ^23^, ^24^, ^25^, ^26^, ^27^, ^28^ ^]^ To gain FDA clearance for sale as surgical masks and surgical N95 respirators in the U.S. market under the typical 510(K) route, the manufacturers should meet the minimum requirements of mask protective ability comprising 1) air filtration capacity, 2) air permeability/differential pressure, 3) fluid resistance, and 4) flammability testing.^[^ ^29^ ^]^ The filtration capacity of a mask is evaluated by measuring its bacterial filtration efficiency (BFE) and particle filtration efficiency (PFE). The BFE test assesses the effectiveness of a mask in filtering bacterial aerosols of 3 µm in size while the PFE test measures how well it filters airborne particles such as virus, pollen and dust (0.1 µm in size).^[^ ^11^ ^]^ The air permeability (also known as differential pressure^[^ ^30^ ^]^) determines how breathable a mask is by measuring how easily air passes through from one side to the other.^[^ ^11^ ^]^ The lower the differential pressure of a mask, the more breathable it is for the wearer.^[^ ^11^ ^]^ The fluid resistance of a mask indicates its resistance to penetration by synthetic blood under pressure while the flammability test determines the time of flame spread over the mask.^[^ ^11^ ^]^

Face masks approved by the FDA can be categorized into three levels based on their performance under the American Society for Testing Materials (ASTM) F2100‐19 standards.^[^ ^31^ ^]^ At level 1, the BFE and PFE should be ≥95% with a differential pressure of <5.0 mmH_2_O cm^−2^. Both level 2 and level 3 require filtration efficiencies of ≥98% and a differential pressure of <6.0 mmH_2_O cm^−2^. Masks under level 1 classification can resist fluid pressure at 80 mmHg, while level 2 and level 3 are at 120 and 160 mmHg, respectively. Although disinfected masks are no longer in their pristine state, public health organizations still maintain the importance of fulfilling these high protective standards in the disinfected masks.^[^ ^9^, ^21^, ^32^ ^]^

Building on the works of others and considering the decontamination guidelines of public health agencies, we investigated the killing efficiencies of UV‐C irradiation, dry heat in static air, and steam sterilization against three types of microorganisms, bacteria (*Staphylococcus aureus*), fungi (*Candida albicans*) and a nonpathogenic virus (*Salmonella virus P22*). Five types of commercially available protective face masks were studied: 1 model of surgical mask (non‐FDA cleared, Faith Guard), 1 model of NIOSH‐approved N95 respirator (Honeywell H801) and 3 models of both NIOSH‐ and FDA‐approved surgical N95 respirators (3M 1860S, 1860, and 1870+). Their BFE, PFE, breathability and fluid resistance were then evaluated based on the standardized test methods recommended by the FDA and the WHO following repeated cycles of disinfection. The effectiveness of each disinfection method was discussed with respect to the FDA bioburden reduction recommendations for reusing decontaminated masks and the ASTM guidelines.

The number of studies evaluating the effectiveness of UV‐C irradiation, dry heat and steam sterilization for mask reusability has increased dramatically since the declaration of the pandemic by WHO in March 2020.^[^ ^23^, ^24^, ^25^, ^26^, ^27^, ^28^, ^33^, ^34^, ^35^, ^36^, ^37^, ^38^, ^39^, ^40^ ^]^ To the best of our knowledge, our study is one of the few that provides a more thorough investigation on the killing efficiency of these methods on masks contaminated with microbes and the performance of these masks following repeated cycles of disinfection. In particular, we have tested the following mask protective functions 1) BFE, 2) PFE, 3) air permeability, and 4) fluid resistance. The findings in this study will provide insights for healthcare officials to better formulate disinfection protocols in line with the approved standards.

## 2 Results and Discussion

The disinfection efficiency of surgical masks and N95 respirators was investigated by using various disinfecting methods: 1) UV‐C irradiation in a commercially purchased UV sterilizer with the UV‐C lamps installed at the top, and UV‐C lamps in a biosafety hood, 2) dry heat in an oven at elevated temperatures between 70 and 100 °C, and 3) steam sterilization with a commercially available steamer. These methods are user friendly and can be potentially employed on a large‐scale basis in hospitals and healthcare settings.

A face mask, if worn properly, is intended to block respiratory droplets from the wearer to the public.^[^ ^8^ ^]^ Therefore, the inner layers (wearer side) of Faith Guard surgical masks and Honeywell H801 N95 respirators were contaminated with *Staphylococcus aureus*, *Candida albicans*, and *Salmonella virus P22*, and disinfected with the abovementioned methods. The assessment on disinfection of surgical N95 respirators for healthcare providers would entail a more stringent approach as they are at higher risks of exposing to infectious microbes than those working in the nonhealthcare sectors. 1860S, 1860, and/or 1870+ from 3M company were selected as the representative surgical N95 respirators worn by healthcare personnel in Singapore. We investigated the effectiveness of the disinfection methods against the microbes contaminated on both the wearer side and the outer layer of these masks. The inclusion of both layers in our study also addresses one of the limitations in numerous published disinfection studies.^[^ ^26^, ^27^, ^33^, ^34^, ^35^ ^]^

**Tables** **1** **2**, and **3** summarize the killing efficiencies against the microbes using UV‐C treatment, dry heat with oven, and steam sterilization, respectively.

**Table 1:** Killing efficiency of microbes contaminated on the inner layers of surgical masks and N95 respirator Honeywell H801 using UV‐C irradiation. Power of UV‐C lamps: 4 W per lamp (×2) in UV sterilizer; 30 W per lamp (×1) in biosafety cabinet

Type/brand of masks	Microbes contamination	UV‐C irradiation conditions	Detection limit [%]	Killing efficiency [%]
Surgical/Faith Guard	*Staphylococcus aureus*	UV sterilizer; 10 min at the contaminated side of mask	>99.9999	>99.9999
		UV sterilizer; 10 min at 180° away from the contaminated side of mask	>99.9999	>99.9999
	*Candida albicans*	UV sterilizer; 10 min at the contaminated side of mask	>99.9999	>99.9999
		UV sterilizer; 10 min at 180° away from the contaminated side of mask	>99.9999	>99.9999
	*Salmonella virus P22*	UV sterilizer; 10 min at the contaminated side of mask	>99.9999	>99.9999
		UV sterilizer; 10 min at 180° away from the contaminated side of mask	>99.9999	99.96 ± 0.04
N95/Honeywell H801	*Staphylococcus aureus*	UV sterilizer; 10 min at the contaminated side of mask	99.923	73.37
		UV sterilizer; 10 min at 180° away from the contaminated side of mask	99.923	50.61
		UV sterilizer; 10 min each at both sides of mask	99.955	70.16
		Biosafety cabinet; 20 min at the contaminated side of mask	99.932	57.93
		Biosafety cabinet; 20 min at 180° away from the contaminated side of mask	99.932	47.74
		Biosafety cabinet; 20 min each at both sides of mask	99.903	63.52

**Table 2:** Killing efficiency of microbes on N95 respirators using dry heat in an oven. All killing efficiencies >99.9999% for *Staphylococcus aureus* and *Candida albicans*, and >99.9% for *Salmonella virus P22* were measured and repeated in triplicate. N.D.: not determined

Type/brand of N95 respirators	Microbes contamination	Dry heat conditions	Detection limit [%]	Killing efficiency at wearer side [%]	Killing efficiency at outer side [%]
H801 Honeywell	*Staphylococcus aureus*	70 °C, 30 min	99.969	94.61	N.D.
		70 °C, 40 min	99.966	94.08	
		80 °C, 40 min	99.966	97.36	
		100 °C, 40 min	99.952	99.904	
			>99.9999	>99.9999	
	*Candida albicans*	100 °C, 40 min	>99.9999	>99.9999	N.D.
	*Salmonella virus P22*			99.97 ± 0.02	
1860S 3M	*Staphylococcus aureus*	100 °C, 1 h	>99.9999	99.9881	99.9784
		100 °C, 2 h		99.9998	99.9988
		100 °C, 3 h		>99.9999	99.9986
	*Candida albicans*	100 °C, 3 h		99.8804	99.6114
	*Salmonella virus P22*	100 °C, 1 h		99.9515	99.88777
		100 °C, 2 h		99.9358	99.51895

**Table 3:** Killing efficiency of microbes on N95 respirators using steam sterilization measured in triplicate. N.D.: not determined

Type/brand of N95 respirators	Microbes contamination	Steam durations	Detection limit [%]	Killing efficiency at wearer side [%]	Killing efficiency at outer side [%]
H801 Honeywell	*Staphylococcus aureus*	5 or 10 min	>99.9999	>99.9999	N.D.
	*Candida albicans*				
	*Salmonella virus P22*				
1860S, 1860, and 1870+ 3M	*Staphylococcus aureus*	5 or 10 min	>99.9999	>99.9999	>99.9999
	*Candida albicans*				
	*Salmonella virus P22*				

UV‐C irradiation for 10 min in the UV sterilizer was effective against surfaces of surgical masks spiked with *Staphylococcus aureus*, *Candida albicans*, and *Salmonella virus P22* with a killing efficiency of >99.9999% (i.e., >6‐log). This complies with the recommendations of FDA to achieve at least 6‐log reductions for vegetative bacteria and nonenveloped viruses under the Tier 2 level of decontamination for reusing masks.^[^ ^32^ ^]^ Noteworthily, for *Staphylococcus aureus* and *Candida albicans*, the same result was demonstrated when the other side of the mask was exposed to the UV‐C (i.e., side of the mask spiked with microbes was faced 180° away from the UV lamps) (Table 1). This result suggests that direct exposure of UV rays to surfaces contaminated with *Staphylococcus aureus* and *Candida albicans* was not necessary and hence, could reduce the time and energy required to disinfect the surgical masks.

For *Salmonella virus P22*, however, a 3.5‐log reduction was observed when the viral contaminated surface was irradiated at 180° away from the UV‐C lamps (Table 1). *Salmonella virus P22* is a nonenveloped bacteriophage with its hosts being *Salmonella typhimurium*.^[^ ^41^ ^]^ The lower killing efficiency is thus not unexpected because nonenveloped viruses are known to be more resistant to environmental changes than other microbes.^[^ ^42^ ^]^ Enveloped viruses such as Coronaviruses and influenza are less environmentally stable than nonenveloped viruses,^[^ ^42^ ^]^ and hence we postulate that UV‐C irradiation might yield a higher killing efficiency against these viruses under the same treatment conditions.

**Figure 1:**
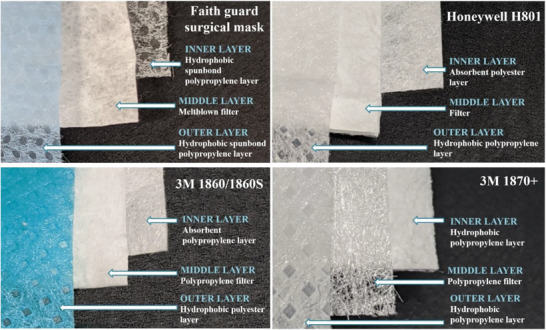
Structure of disposal nonwoven face masks, Faith Guard surgical mask, and N95 respirators (Honeywell H801, 3M 1860/1860S, and 3M 1870+). The inner layers of Honeywell H801 and 3M 1860/1860S absorb water while their outer layers repel water. Both layers of Faith Guard surgical mask and 3M N95 1870+ repel water.

On the other hand, UV‐C irradiation of N95 respirator Honeywell H801 using the UV sterilizer did not perform well. When irradiated at the contaminated side for 10 min, only 73.4% of *Staphylococcus aureus* was inactivated. Irradiating at both sides for 10 min each produced a comparable killing efficiency of 70.2%. UV exposure at the uncontaminated side for 10 min, however, could only inactivate 50.6% of the viable bacteria. Irradiation under UV‐C lamp for 20 min in the biosafety cabinet was not effective either. The killing efficiency of UV‐C irradiating at the uncontaminated side, the contaminated side, and both sides was 47.7%, 57.9%, and 63.5%, respectively (Table 1). The vast difference in the killing efficacy between surgical masks and N95 respirators may be attributed to the thickness and hydrophobicity of the inner layer. Both the inner and outer layers of the Faith Guard surgical mask appear to be hydrophobic as indicative by the immediate repelling of microbial inoculum (**Figure** **1**). The inner side of the Honeywell H801 N95 respirator, on the other hand, is a thick porous mesh and absorbs the microbial inoculum (Figure 1). The fibrous network of the mask potentially provided shadowing effects to the microbial cells which helped them stay hidden from the UV irradiation. This consequently resulted in a poorer killing efficiency as the UV‐C light was unable to penetrate through the mesh to reach the hidden cells.^[^ ^43^ ^]^ A relatively lower log reduction due to shadowing effects in a N95 respirator model was also reported in a paper by Mills et al.^[^ ^34^ ^]^ Nevertheless, UV‐C treatment is an effective disinfection method for surgical masks. It is also a well‐accepted method for certain types/models of N95 respirators and has already been put into practice by some institutions during the COVID‐19 pandemic.^[^ ^23^ ^]^

Thermal inactivation of microorganisms is another well‐established sterilizing method involving an inverse relationship between temperature and exposure times.^[^ ^44^ ^]^ Depending on the types of microbes being tested, previous studies have demonstrated varying effectiveness of dry heating at temperatures ranging from 60 to 100 °C for a duration between 30 min and 1 h.^[^ ^36^, ^37^, ^38^, ^39^, ^40^, ^44^ ^]^ For instance, a recent study showed that dry heat at 70 °C for 30 min in an electric oven was insufficient to achieve a 99.9% killing efficiency against *Staphylococcus aureus*.^[^ ^36^ ^]^ Another recent study revealed that the inactivation efficiency of dry heating at 82 °C for 20 min using an industrial washer was less than 90% for *Staphylococcus aureus*.^[^ ^37^ ^]^ These align with our findings where only ∼94% and 97.4% of the bacteria was inactivated at the inner layer of Honeywell H801 N95 respirator when the mask was treated with dry heat at 70 °C for 30–40 min and 80 °C for 40 min, respectively (Table 2). When the temperature was elevated to 100 °C, an exposure time of 40 min inactivated 99.9% of the bacteria (Table 2). To further challenge this condition, the inner layer of the masks was contaminated with an increased bacterial count by ≈3‐log to 10^6^ CFU mL^−1^. Dry heat at 100 °C for 40 min was able to kill >99.9999% of the *Staphylococcus aureus*. This finding indicated that heat conduction within the mesh offered a deeper penetration than UV‐C irradiation, which in turn contributed to a much higher killing efficacy. Additionally, this condition could reduce the viability of *Candida albicans* and *Salmonella virus P22* by >6‐log and >3‐log, respectively (Table 2).

**Figure 2:**
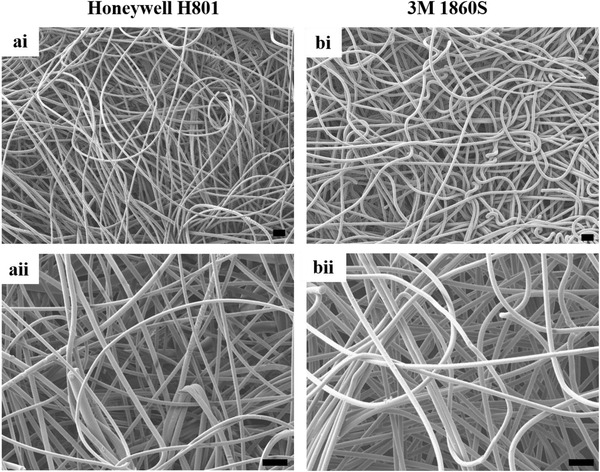
Scanning electron microscopy of the inner layer of N95 respirators. a) Honeywell H801 and b) 3M 1860S. The i) top panel and ii) bottom panel represent images at magnification of 50× and 100×, respectively. Scale bars: 100 µm.

Dry heat at 100 °C for 40 min worked effectively as a disinfection method against the microbes tested on the inner layer of Honeywell H801. On the inner layer of 3M 1860S, however, the same treatment condition did not meet the minimum requirements of 6‐log reduction for vegetative bacteria under the Tier 2 system of FDA bioburden reduction recommendations.^[^ ^32^ ^]^ A longer duration of 3 h at 100 °C was required to achieve >99.9999% killing efficiency for *Staphylococcus aureus* (Table 2). This vast difference in the duration between the two mask models may be explained by the fabric thickness and density of the inner layer. The material comprising the inner layer of 3M 1860S is polypropylene and has a higher intrinsic thermal conductivity than the polyester in Honeywell H801.^[^ ^45^ ^]^ However, the thickness of 3M 1860S inner layer is approximately 1.7‐fold higher. This provided a better thermal insulation and could possibly offset its material thermal conductivity.^[^ ^45^ ^]^ Additionally, the fabric density of the inner surface was evaluated through scanning electron microscopy. The inner layer of Honeywell H801 is more porous and hence has a lower fabric density than 3M 1860S (**Figure** **2**). For nonwoven fabrics, the thermal conductivity has an inverse correlation with the fabric density.^[^ ^46^ ^]^ Coupled with its smaller thickness, the inner layer of Honeywell H801 might correspondingly conduct heat more efficiently than 3M 1860S.

Dry heat at 100 °C for 3 h, however, did not yield the same result of >99.9999% killing efficiency for *Candida albicans* (Table 2). The killing efficiencies at the outer layer were generally lower than that of the inner layer across all microbes tested (Table 2) possibly due to the lower intrinsic thermal conductivity of polyester,^[^ ^45^ ^]^ which was used to make the outer layer of the mask (Figure 1). To illustrate, >3‐log reduction was achieved at the inner layer contaminated with *Salmonella virus P22*, while the reduction at the outer layer was <3‐log after dry heating at 100 °C for 1–2 h. We did not attempt higher temperatures as dry heat above 100 °C may affect the structural integrity of the masks.^[^ ^38^ ^]^ Collectively, findings of dry heating have highlighted the importance of optimizing the disinfection conditions for different types/models and different layers of the masks being tested.

In contrast, moist heat in the form of steam is an effective disinfection method for both Honeywell H801 and 3M N95 respirators against the bacteria, yeast and virus. Steam sterilization for 5 or 10 min was able to achieve >99.9999% killing efficiencies across all tested microbes, including *Salmonella virus P22*, for Honeywell H801 and the three 3M N95 models at both the inner and outer layers (Table 3). Steam sterilization is a practical method due to its low contact time and ease of accessibility to the public. However, this method may be not be suitable for decontaminating surgical masks due to loss of protective capability of the masks after the disinfection.^[^ ^36^, ^39^, ^40^ ^]^

In the event of unexpected shortages of face masks especially during the initial phase of the COVID‐19 pandemic, the U.S. CDC recommends a limited reuse of N95 respirators under the Crisis Capacity Strategies.^[^ ^21^ ^]^ To supplement the existing CDC reuse recommendations of N95 decontamination, the FDA maintains a bioburden reduction of a nonenveloped virus or vegetative bacteria by ≥3‐log under the Tier 3 level of decontamination.^[^ ^32^ ^]^ Based on our findings, disinfection using dry heat (100 °C, ≥40 min) on Honeywell H801, and steam sterilization (≥5 min) on all the four N95 respirators would fulfill this requirement. Under the Tier 2 level of decontamination, the disinfection method should yield ≥6‐log reduction of nonenveloped viruses or vegetative bacteria.^[^ ^32^ ^]^ With this stricter guideline, only UV‐C treatment of Faith Guard surgical masks (10 min per side to ensure thorough disinfection) and steam sterilization of the N95 respirators (≥5 min) are satisfied.

Additionally, the U.S. CDC and ECDC recommend that the decontamination methods should not physically damage or degrade the masks.^[^ ^21^, ^22^ ^]^ Visual inspection of the face masks showed no observable deformation before and after 10 cycles of UV‐C irradiation and dry heating. Following 1 cycle of steam sterilization, the original label on the outer surface of both 3M 1860S and 3M 1860 masks smudged considerably while the label on 3M 1870+ and Honeywell H801 remained visible. Smudging of label after the steam sterilization may be resolved by pre‐labelling with water resistant label which do not affect the function of the masks. No other noticeable deformation of the masks was observed after the steam treatment.

Repeated exposure to UV radiation and high temperatures from heat as well as moisture from steam may, however, affect the integrity of the masks microscopically which could impair their overall performance.^[^ ^39^, ^40^ ^]^ We next assessed the filtration efficiencies and breathability of the masks with respect to the number of disinfection cycles. For 3M 1860S, 1860, and 1870+ surgical N95 respirators, their resistance to synthetic blood penetration was also investigated. Note that this testing criterion is not mentioned in the current FDA recommendations for seeking pre‐Emergency Use Authorization for decontamination of FDA‐cleared surgical masks and N95 respirators.^[^ ^32^ ^]^ The three models of surgical 3M N95 respirators are FDA‐cleared, but fluid resistance test was included in our study since the disinfection process may affect their ability to resist fluid splash. This performance evaluation is especially important as healthcare personnel are at risks of exposing to infectious microorganisms through splash of bodily fluids from infected patients. **Table** **4** summarizes the performance outcome in terms of BFE, PFE, air permeability/differential pressure as a measurement for breathability, and fluid resistance after various treatments.

**Table 4:** Performance of face masks over number of disinfection cycles. BFE: Bacteria Filtration Efficiency; PFE: Particle Filtration Efficiency (N.D.: not determined. BFE was performed in accordance with the ASTM F2101‐19 standard test method with *Staphylococcus aureus* as the aerosol bacterial challenge with mean particle size of 2.9 ± 0.5 µm. PFE was performed in accordance with the ASTM F2299 standard test method with 0.1 µm size polystyrene latex particle aerosol. The air permeability/differential pressure across a mask sample was evaluated according to the EN14683‐2019 standard. The fluid resistance of a mask sample was conducted according to the ASTM F1862/F1862M‐17 test method. PFE of 3M 1870+ N95 masks was not determined as the size of the masks was too small to cover the testing area)

Mask/Brand	Disinfection	Number of cycles	BFE [%]	PFE [%]	Differential pressure (mmH_2_O cm^−2^)	Fluid resistance
Surgical mask/Faith Guard	Original		99.6 ± 0.2	96.1 ± 0.5	5.6 ± 0.7	N.D.
	UV sterilizer, 10 min per side (both sides)	10	99.6 ± 0.2	96.1 ± 0.5	5.6 ± 0.1	
N95/Honeywell H801	Original		99.95^a)^	99.8 ± 0.0	7.0 ± 0.4	N.D.
	Dry heat at 100 °C for 40 min	5	99.95^a)^	99.8 ± 0.0	7.0 ± 0.5	
		10	99.95^a)^, ^b)^	99.6 ± 0.1	6.8 ± 0.3	
	Steam for 10 min	5	99.8 ± 0.1	98.7 ± 0.6	6.4 ± 0.1	
		10	99.9 ± 0.1	97.0 ± 0.5	6.4 ± 0.7	
N95/3M 1860S	Original		99.94^a)^	99.7 ± 0.1	5.2 ± 0.4	80 mmHg (pass:3/total:3)
	Steam for 10 min	5	99.94^a)^	99.6 ± 0.2	5.6 ± 0.1	
N95/3M 1860	Original		99.93^a)^	99.6 ± 0.1	5.4 ± 0.1	120 mmHg (pass:3/total:3)
	Steam for 10 min	5	99.93^a)^	99.6 ± 0.1	5.8 ± 0.2	
N95/3M 1870+	Original		99.96^a)^	N.D.	6.8 ± 1.0	160 mmHg (pass:3/total:3)
	Steam for 10 min	5	99.92^a)^	N.D.	6.5 ± 0.7	

^a)^ indicates detection limit being hit

^b)^ Results obtained from duplicates.

Faith Guard surgical masks were irradiated with UV‐C in the UV sterilizer for 10 min each on the wearer layer and the outer layer (1 cycle). The BFE and PFE of these surgical masks remained at >99.9% and >96% after 10 cycles, respectively. The differential pressure of these masks remained unchanged after 10 cycles of UV‐C treatment. In contrast to surgical masks, N95 respirators have thicker three‐dimensional structure which requires higher doses of UV‐C irradiation for effective disinfection. This, however, leads to mask degradation over time.^[^ ^39^ ^]^ To prolong the reusability of masks, surface coating with UV‐resistant materials may be explored.^[^ ^47^ ^]^ The BFE and PFE of untreated Honeywell H801 were ≥99.9% and 99.6%, respectively. Following 10 cycles of dry heating at 100 °C for 40 min per cycle, the filtration efficiencies did not vary significantly (Table 4). Additionally, the breathability of the masks was comparable to the untreated ones. For steam sterilization, the upper duration limit of 10 min was tested since a longer cycle of treatment is associated with a more detrimental impact on mask performance.^[^ ^28^ ^]^ The PFE of Honeywell H801 fell below the minimum level 2 (level 3) ASTM F2100‐19 standard requirement of 98% after 10 cycles of steam treatment. This demonstrates that steam has a higher destructive impact to the integrity of masks than dry heat. Nonetheless, steam sterilization of Honeywell H801 for 10 min per cycle exhibited ≥98% for both BFE and PFE at the 5th cycle.

The filtration efficiencies (BFE and PFE) of 3M 1860S, 1860, and 1870+ surgical N95 respirators were maintained at ≥99% after 5 cycles of steam treatment at 10 min per cycle. The rise of differential pressure for 3M 1860S and 1860 surgical N95 respirators following 5 cycles of steam sterilization was insignificant and remained under 6.0 mmH_2_O cm^−2^. In addition, all three pieces per model of the surgical N95 respirators from 3M passed the fluid resistance test after 5 cycles of steam treatment.

## 3 Conclusion

UV‐C irradiation for 10 min in a commercial UV sterilizer was effective to disinfect surgical masks while maintaining the filtration efficiencies for up to 10 cycles. N95 respirator Honeywell H801 required thermal inactivation with dry heat up to 100 °C for 40 min to achieve >99.9999% killing efficiency against bacteria, yeast, and virus without compromising the BFE, PFE, and breathability for up to 10 cycles. Dry heat at 100 °C for up to 3 h did not yield >99.9999% killing efficiency against yeast and virus for 3M N95 masks. Steam sterilization, on the other hand, only required a short duration of 5 min to achieve a killing efficiency of at least 6‐log reduction against all the microbes tested for Honeywell H801 and the 3M surgical N95 respirators (1860S, 1860, and 1870+). More importantly, 5 cycles of steam treatment (10 min per cycle) did not impair their bacteria and particle filtration capabilities, breathability, and fluid resistance property. The findings of this study will be beneficial to formulating standard procedures for a safe reuse of surgical masks and N95 respirators through commonly accessible disinfection methods.

## 4 Experimental Section

### Materials

All materials were used as received unless otherwise specified. Disposable nonwoven 3‐ply surgical masks were procured from Faith Guard (Singapore). Disposable nonwoven N95 respirators Honeywell H801 and 3 models of surgical N95 respirators, 1860S, 1860, and 1870+, were obtained from Honeywell (Singapore) and SingHealth (Singapore), respectively. Note that 3M 1860S and 1860 only differ in the size (1860S is a smaller version of 1860) and fluid resistance. *Staphylococcus aureus* (ATCC No. 6538), yeast *Candida albicans* (ATCC No. 1023), and *Salmonella typhimurium* (ATCC No. 14 028) were obtained from ATCC (U.S.A.) and reconstituted according to supplier's instructions. *Salmonella virus P22* was a generous gift from Prof. Linda J Kenney in the Mechanobiology Institute, National University of Singapore. Cation‐adjusted Mueller‐Hinton broth (MHB) powder was purchased from BD Diagnostics (Singapore) and used as broth for culturing the microbes. LB agar powder at 2.5% LB and 1.5% agar was obtained from Biotech (Singapore).

### Sample Preparation

The surgical masks and N95 respirators were cut into pieces of 5 cm × 5 cm and 2 cm × 2cm, respectively, prior to the disinfection studies. Suspensions of *Staphylococcus aureus* and *Candida albicans* were cultured in MHB at 37 °C and room temperature, respectively, under constant shaking at 300 rpm. Following overnight culturing, the microbial suspensions were subsequently added to either sides of the cut mask pieces at random to give a colony forming units (CFU) ranging from 0.8 × 10^2^ to 2.5 × 10^3^ per piece. An enhanced microbial challenge was performed with an increased microbial load of >10^6^ CFU mL^−1^. *Salmonella virus P22* was added with a viral load of 1.3 × 10^7^–6.8 × 10^7^ plaque forming units (PFU) per mL. Microbes were added to the inner (wearer) layer of Faith Guard surgical mask and N95 respirator Honeywell H801, while 3M surgical N95 respirators 1860S, 1860, and 1870+ were contaminated at either the inner (wearer) or outside layer. The inner layer of Honeywell H801 and 3M 1860/1860S absorb water while the outer layer repels water. Both layers of Faith Guard surgical mask and 3M N95 1870+ repel water.

### Ultraviolet‐C (UV‐C) Irradiation

Samples were exposed to UV‐C radiation by placing them into a UV sterilizer chamber (Hanil UV Multi Sterilizer Dryer), where two UV‐C lamps (OSRAM, 4W) were installed at the top or under an UV‐C lamp (Philips TUV30W G30T8 germicidal fluorescent light bulb) in a biosafety cabinet (LabGard AIR model NU‐543‐400S class II) for various durations and cycles. Both UV‐C lamps in the UV sterilizer chamber and the biosafety cabinet operate at a wavelength of 253.7 nm. After the UV‐C‐ treatment, samples were transferred into a tube of MHB (5 mL), vortexed for 5 min and removed from the broth. Microbes in the broth were then plated on agar and incubated at 37 °C overnight for counting colonies. Samples unexposed to UV radiation were used as controls.

### Dry Heat in Static Air

Samples were exposed to static air ranging from 70 to 100 °C in a vacuum oven (MMM Medcenter Einrichtungen GmbH Vacucell 55) over various durations. The vacuum oven was under atmospheric pressure throughout the course of study. A glass thermometer was placed inside the oven to ensure accurate temperature readings. Following the hot air exposure, the samples were processed as described in the above section. Broth containing *Candida albicans* was plated on agar and incubated at room temperature for 48 h before counting the CFU. Broth containing *Salmonella virus P22* was plated on soft agar premixed with *Salmonella typhimurium* and incubated at 37 °C overnight before counting the PFU. Samples unexposed to the hot air were used as controls.

### Steam Sterilization

Samples were placed in a steamer (Philips HD9125/01) and steamed for 5 and 10 min. They were then processed accordingly as described in the sections of *Ultraviolet‐C (UV‐C) irradiation* and *Dry heat in static air*. Samples unexposed to steam were used as controls.

### Killing Efficiency

The killing efficiency of a disinfection method against the microbes was determined as follows

(1) $$\begin{array}{c} \begin{array}{l} \text{Killing}\;\text{efficiency}\left(\%\right)=\\ \frac{\text{CFU}\;\text{or}\;\text{PFU}\;\text{in}\;\text{control}-\text{CFU}\;\text{or}\;\text{PFU}\;\text{in}\;\mathrm{disinfected}\;\text{sample}\;}{\text{CFU}\;\text{or}\;\text{PFU}\;\text{in}\;\text{control}}\times 100\% \end{array} \end{array}$$

Any disinfection method which achieved a killing efficiency of ≥99.9999% was repeated twice to check for reproducibility.

### Scanning Electron Microscopy

The structural network of the inner layer of N95 respirators was imaged using a scanning electron microscopy at 5kV (JSM‐7400F, JEOL).

### Bacteria Filtration Efficiency (BFE)

**Scheme 1:**
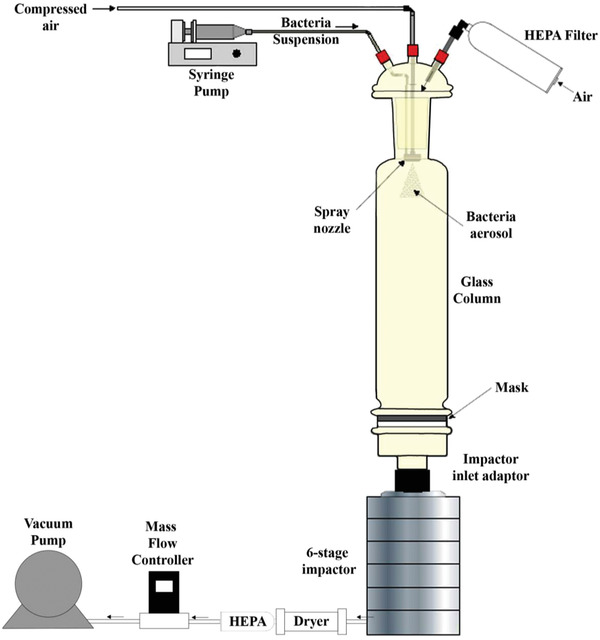
A schematic setup to test the BFE in accordance with the ASTM F2101‐19 Standard Test Method. A liquid suspension of *Staphylococcus aureus* was filled into a syringe and drawn by the pump into the spray nozzle which was emitted as aerosol (mean particle size: 2.9 ± 0.5 µm) at a constant flow rate of 28.3 L min^−1^_._ Mask samples were placed at the bottom of the glass column with a test area of 38.5 cm^2^ before the start of bacterial aerosolization. With the suction from vacuum pump, the aerosolized bacteria passed through the glass column towards the 6‐stage impactor containing agar plates. The agar plates were subsequently incubated overnight at 37 °C for colony counting. All mask samples were tested in triplicate with the outer layer facing the bacterial aerosol.

The bacterial filtration efficiency of the samples was measured in accordance with the ASTM F2101‐19 Standard Test Method for Evaluating the Bacterial Filtration Efficiency (BFE) of Medical Face Mask Materials (**Scheme** **1**). A biological aerosol of *Staphylococcus aureus* was used as the bacterial challenge at a constant flow rate of 28.3 L min^−1^. Mask samples to be tested were placed at the bottom of the glass column with a test area of 38.5 cm^2^. All mask samples were tested with the outer layer facing the bacterial challenge (i.e., BFE with respect to protecting the wearer). Bacterial aerosolization without masks at the bottom of the glass column was used as controls. Agar plates were placed inside the 6‐stage impactor prior to the start of bacterial aerosolization and incubated overnight at 37 °C for colony counting. The mean particle size of the challenge aerosol was maintained at 2.9 ± 0.5 µm. BFE of three samples was measured to obtain the average BFE using the following equation

(2) $$\begin{array}{c} \text{BFE}\left(\%\right)=\frac{\text{CFU}\;\text{of}\;\text{control}-\text{CFU}\;\text{of}\;\text{samples}\;}{\text{CFU}\;\text{of}\;\text{control}}\times 100\% \end{array}$$

### Particle Filtration Efficiency (PFE)

The PFE of a mask sample was tested according to ASTM F2299 by penetration of 0.1 µm polystyrene latex spheres. The effective sample size was about 45.6 cm^2^ and the flow rate was controlled around 28.3 L min^−1^. PFE was calculated from the particulate concentrations of upstream and downstream with the following equation

(3) $$\begin{array}{c} \begin{array}{l} \text{PFE}\left(\%\right)=\\ \frac{\text{Upstream}\;\text{Concentration}-\text{Downstream}\;\text{Concentration}\;}{\text{Upstream}\;\text{Concentration}}\times 100\% \end{array} \end{array}$$

### Air Permeability/Differential Pressure Test

The air permeability/differential pressure across a mask sample was determined according to EN14683‐2019 standard. The inner diameter of the tube was 1.0 inch and the air flow rate was adjusted to 8.0 L min^−1^ through a diaphragm valve. The differential pressure was measured using a differential pressure gauge.

### Fluid Resistance Test

The fluid resistance of a mask sample was evaluated according to ASTM F1862/F1862M‐17 by horizontally projecting a small volume (≈2 mL) of synthetic blood with a pre‐determined velocity. The samples were conditioned in a humidity chamber for at least 4 h by exposure to a temperature of 21 °C and a relative humidity of 85% before testing. The mask samples were tested with the outer layer facing the high velocity fluid corresponding to the respective fluid pressure according to the manufacturer's product specifications. The penetration of synthetic blood to the inner layer of a mask was inspected after 10 s from the stop of projection.

## Conflict of Interest

The authors declare no conflict of interest.

## Author Contributions

J.Y.T., B.P., and Y.Y.Y. designed the experiments. J.Y.T., J.K., and B.P. performed the disinfection studies and the bacterial filtration efficiency study. S.L., P.C., and X.N. performed the particle filtration efficiency and differential pressure experiments. G. Y. and Y.Z. conducted the scanning electron microscopy. C.E.L., B.H.T., D.T., Y.Y.O., M.L.L., W.Y.W., H.M.W., M.H., and X.X. provided clinical insights and reviewed the experimental designs. All authors participated in the analysis of the experimental results. J.Y.T. and J.K. drafted the manuscript with inputs from all authors. Y.Y.Y. supervised the study and edited the manuscript.

## Data Availability

Research data are not shared.
